# Prognostic value of post-treatment serum soluble interleukin-2 receptor in newly diagnosed diffuse large B-cell lymphoma patients who achieved complete metabolic response following R-CHOP therapy

**DOI:** 10.1038/s41598-023-40026-7

**Published:** 2023-08-22

**Authors:** Yuko Shirouchi, Noriko Nishimura, Yuko Mishima, Yuko Ishihara, Hiroaki Asai, Mikako Tamba, Mitsuhito Hirano, Kei Hirano, Yukako Teramoto, Kikuaki Yoshida, Kengo Takeuchi, Takashi Terauchi, Dai Maruyama

**Affiliations:** 1 Department of Hematology Oncology, Cancer Institute Hospital, Japanese Foundation for Cancer Research, 3-8-31Ariake, Koto, Tokyo, Japan; 2 Division of Pathology, The Cancer Institute, Japanese Foundation for Cancer Research, Tokyo, Japan; 3 Pathology Project for Molecular Targets, The Cancer Institute, Japanese Foundation for Cancer Research, Tokyo, Japan; 4 Department of Pathology, Cancer Institute Hospital, Japanese Foundation for Cancer Research, Tokyo, Japan; 5 Department of Nuclear Medicine, Cancer Institute Hospital, Japanese Foundation for Cancer Research, Tokyo, Japan

**Keywords:** Haematological cancer, Lymphoma, Non-hodgkin lymphoma, B-cell lymphoma

## Abstract

Patients with DLBCL achieving complete metabolic response (CMR) after initial treatment with R-CHOP generally have a favourable prognosis; however, there are no established prognostic biomarkers for relapse in these patients. Soluble interleukin-2 receptor (sIL-2R) levels at diagnosis are prognostic factors in patients with DLBCL. However, the significance of post-treatment sIL-2R levels is unclear. To determine the significance of post-treatment serum sIL-2R levels on subsequent relapse and survival, we retrospectively analysed 485 patients with newly diagnosed DLBCL who received R-CHOP treatment and achieved CMR. The cumulative incidence of relapse (CIR) was significantly higher in patients with elevated post-treatment sIL-2R levels than in those with normal sIL-2R levels (five-year CIR; 38.8% vs. 12.8%). The prognostic value remained significant in multivariable analysis (hazard ratio, 2.30; *p* < 0.001). Five-year progression-free survival (49.0% vs. 83.5%) and overall survival (61.7% vs. 91.6%) rates were lower in patients with elevated post-treatment sIL-2R levels than in those with normal sIL-2R levels (*p* < 0.001 for both). In patients with newly diagnosed DLBCL who achieved CMR after R-CHOP treatment, the post-treatment serum sIL-2R level was an independent prognostic marker of subsequent relapse and survival.

## Introduction

Diffuse large B-cell lymphoma (DLBCL) is the most common subtype of lymphoma, accounting for approximately 30% of all lymphomas^1,2^. For the past two decades, R-CHOP (rituximab, cyclophosphamide, doxorubicin, vincristine, and prednisolone) has been the standard therapy for newly diagnosed DLBCL^3,4^. Patients who achieve complete response after R-CHOP generally show a favourable prognosis^5–7^, and although approximately 60% of patients maintain long-term response after R-CHOP-like therapy and are possibly cured, the remaining 40% experience disease progression, resulting in a dismal prognosis^8,9^. Therefore, early identification of the patients who will show disease progression is necessary to improve their outcomes. However, there are no established biomarkers to identify patients who show a complete response but with a high risk of relapse. The International Prognostic Index (IPI) is a widely used risk-stratification model for non-Hodgkin lymphoma^10^, however, it may not sufficiently discriminate between risk groups in the rituximab era^11^.

Soluble interleukin-2 receptor (sIL-2R) is the soluble form of the interleukin-2 receptor. The interleukin-2 receptor consists of three glycoprotein chains: α, β, and γ^12,13^. IL-2R is expressed on lymphocyte membranes and plays a role in their activation and proliferation^13^. When the α subunit of IL-2R is cleaved from the cell membrane and released into the serum, it is detected as sIL-2R^14^. sIL-2R levels are elevated in patients with malignant lymphomas, including DLBCL. Several studies have suggested that the sIL-2R level at diagnosis has prognostic significance for survival in patients with DLBCL^15–17^. However, little is known about the significance of post-treatment serum sIL-2R levels. In this study, we aimed to determine the value of post-treatment serum sIL-2R levels on subsequent relapse, progression-free survival (PFS), and overall survival (OS) in patients with DLBCL who achieved complete metabolic response (CMR) following R-CHOP therapy.

## Methods

### Patient population

Patients newly diagnosed with DLBCL between January 2005 and December 2016 at our institution who were treated with R-CHOP with or without local radiation therapy (RT) and achieved CMR, as observed with ^18^F-fluorodeoxyglucose positron emission tomography/computed tomography (FDG-PET/CT) were included in this study. Histopathological diagnoses were performed by a haematopathologist at our institution (KT) in accordance with the World Health Organization classification of tumours of haematopoietic and lymphoid tissues^2,18,19^. Patients who achieved partial metabolic response (PMR) after R-CHOP therapy and achieved CMR after additional RT to the remaining lesions were included. Patients who did not undergo end-of-treatment FDG-PET/CT, those with DLBCL transformed from low-grade lymphoma, and those with concomitant low-grade B-cell lymphoma were excluded from the study. The included patients were divided into two groups according to their post-treatment sIL-2R levels: patients with elevated sIL-2R levels (> upper limit of normal [ULN]) and those with normal sIL-2R levels. Data from these groups were retrospectively analysed. The study design was approved by the ethics committee of the Japanese Foundation for Cancer Research before the initiation of this study. This study was conducted in accordance with the international ethical recommendations of the Declaration of Helsinki. The need for informed consent was waived by the above-mentioned ethics committee due to the retrospective nature of the study.

### Assessment

The objective of this study was to determine the relationship between post-treatment serum sIL-2R levels and subsequent relapse, PFS, and OS in patients with newly diagnosed DLBCL who achieved CMR after treatment with R-CHOP with or without local RT. Treatment response was evaluated by the treating physicians using FDG-PET/CT according to the revised response criteria for malignant lymphoma (if the assessment was made before 2014) or the Lugano criteria (if the assessment was made from 2014 onwards)^20,21^. Post-treatment sIL-2R level was defined as the sIL-2 level at the time of FDG-PET/CT evaluation. The cell of origin was determined using the Hans criteria^22^. Enzyme immunoassay was used for serum sIL-2R analysis. Based on the previous studies evaluating sIL-2R in lymphoma^23,24^, the cut-off value for sIL-2R was set as 519 U/mL, which was the official ULN for the testing methods used in this study. Additionally, ROC analysis was performed to determine the original cut-off value for serum sIL-2R for this study cohort.

### Statistical analyses

The cumulative incidence of relapse (CIR) was estimated and compared between groups using Gray’s test. Death due to causes other than lymphoma was considered a competing event. OS and PFS were estimated using the Kaplan–Meier method and compared between groups using the log-rank test. For multivariable analysis, the Fine–Gray model was used to identify the risk factors associated with relapse, and Cox proportional hazards regression was used to identify the risk factors associated with PFS and OS. OS was defined as the time from initial response assessment to death from any cause, and PFS was defined as the time from initial response assessment to disease progression/relapse or death from any cause. All statistical analyses were performed using EZR (Saitama Medical Center, Jichi Medical University, Saitama, Japan)^25^, a graphical user interface for R (The R Foundation for Statistical Computing, Vienna, Austria). *P* < 0.05 was considered to indicate statistical significance.

### Ethical approval

This study was performed in accordance with the Declaration of Helsinki and the ethical standards of the institutional research committee.

### Informed consent

The need for informed consent was waived by the ethics committee of the Japanese Foundation for Cancer Research due to the retrospective nature of the study.

## Results

### Patient characteristics

In total, 485 patients were included in the analysis (elevated sIL-2R levels, n = 105; normal sIL-2R levels, n = 380; Supplementary Figure S1 online). The baseline characteristics of the patients at the time of diagnosis in each group are shown in Table 1. The median pre-treatment sIL-2R level value was 707 U/mL and the median post-treatment sIL-2R level value was 387 U/mL. More patients in the elevated sIL-2R group had B symptoms, high-intermediate or high IPI, elevated pre-treatment IL-2R levels, and advanced-stage disease. All patients were treated with R-CHOP. Ninety-eight patients received RT in combination with R-CHOP; 72 patients received RT as part of the planned treatment, while 26 received additional RT because FDG-PET/CT upon completion of R-CHOP showed PMR. All patients who underwent additional RT achieved CMR after RT. The median follow-up duration was 79 months (range, 7–178 months).

**Table 1:** Patient characteristics.

Factor	Group	post-treatment sIL-2R ≤ UNL (n = 380)	post-treatment sIL-2R > UNL (n = 105)	*P* value
Age	< 65	151 (39.7)	18 (17.1)	< 0.001
	≥ 65	229 (60.3)	87 (82.9)	
B symptoms	Present	49 (12.9)	32 (30.5)	< 0.001
	Absent	331 (87.1)	73 (69.5)	
Bone marrow invasion	Yes	54 (14.2)	19 (18.1)	0.355
	No	326 (85.8)	86 (81.9)	
COO	GCB	207 (55.8)	44 (44.0)	0.042
	Non-GCB	164 (44.2)	56 (56.0)	
Extranodal lesions	< 2	323 (85.0)	73 (69.5)	0.001
	≥ 2	57 (15.0)	32 (30.5)	
IPI	Low/low-int	298 (78.4)	60 (57.1)	< 0.001
	High/high-int	82 (21.6)	45 (42.9)	
PS	0–1	369 (97.1)	95 (90.5)	0.006
	2–4	11 ( 2.9)	10 (9.5)	
Pre-treatment sIL-2R	≤ UNL	156 (41.1)	7 (6.7)	< 0.001
	> UNL	224 (58.9)	98 (93.3)	
Sex	Female	175 (46.4)	43 (41.3)	0.439
	Male	202 (53.6)	61 (58.7)	
Stage	I, II	261 (68.7)	50 (47.6)	< 0.001
	III, IV	119 (31.3)	55 (52.4)	

*COO* cell of origin, *GCB* germinal center B-cell-like type, *high-int* high-intermediate, *IPI* international prognostic index, *low-int* low-intermediate, *PS* performance status, *sIL-2R* soluble interleukin-2 receptor, *UNL* upper normal limit.

### Relapse

**Figure 1:**
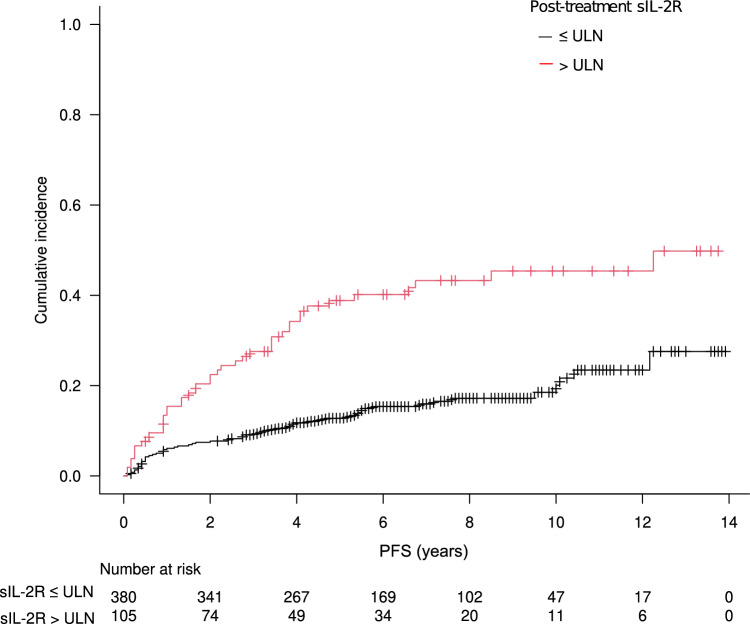
Cumulative incidence of relapse. Significantly higher CIR was observed in the elevated sIL-2R group (five-year CIR, 38.8%; 95% CI, 29.0–48.5%) compared with the normal sIL-2R group (five-year CIR, 12.8%; 95% CI, 9.6–16.5; *p* < 0.001). CIR—cumulative incidence of relapse; sIL-2R—soluble interleukin-2 receptor; ULN—upper limit of normal.

With a median follow-up period of 6.1 years (range, 0.2–14.4 years), CIR at 5 years was 18.4% (95% CI, 15.0%–22.1%) in the overall patient cohort and 38.8% (95% CI; 29.0–48.5%) in the elevated sIL-2R group, which was significantly higher than that in the normal sIL-2R group (12.8%; 95% CI, 9.6–16.5; hazard ratio [HR], 2.93; 95% CI, 1.97–4.35, *p* < 0.001; Fig. 1). The sensitivity of post-treatment sIL-2R levels > ULN in predicting relapse was 41.7% (95% CI, 32.1–51.9) and the specificity was 83.8% (95% CI, 79.7–87.3). The prognostic value of post-treatment sIL-2R group remained significant when an analysis was limited to those had elevated pre-treatment sIL-2R levels (> ULN), with the five-year CIR of 39.7% (95% CI, 29.4% – 49.8%) for those with elevated post-treatment sIL-2R levels, and 15.9% (95% CI, 11.3%–21.1%) for those with normal post-treatment sIL-2R levels (HR 2.30, 95% CI, 1.50–3.51, *p* < 0.001). Moreover, elevated post-treatment sIL-2R was significantly associated with lower CIR rates in both patients with and advanced stage diseases: In patients with limited-stage disease, the five-year CIR was 21.3% (95% CI, 10.9% – 34.1%) for those with elevated post-treatment sIL-2R levels, and 6.9% (95% CI, 4.2% – 10.5%) for those with normal post-treatment sIL-2R levels (HR 2.82, 95% CI, 1.44–5.55, *p* = 0.003). In patients with advanced-stage disease, the five-year CIR was 55.3% (95% CI, 40.0% – 68.2%) for those with elevated post-treatment sIL-2R levels, and 25.9% (95% CI, 18.0% – 34.4%) for those with normal post-treatment sIL-2R levels (HR 2.17, 95% CI, 1.34–3.52,* p* = 0.002). The CIR did not differ between patients who achieved CMR following R-CHOP (with or without planned RT) and those who achieved CMR after additional RT (five-year CIR, 18.8% vs. 11.9%; *p* = 0.20). The results of the univariable analysis for CIR, including various clinicopathological characteristics at diagnosis known to be prognostic in DLBCL and post-treatment sIL-2R levels, are shown in Supplementary Table S1 online. In multivariable analysis, post-treatment sIL-2R level > ULN remained prognostic of subsequent relapse (HR, 2.30; *p* < 0.001), along with non-GCB type, initial bone marrow invasion, and advanced stage at diagnosis (Table 2, Supplementary Figure S2 online).

**Table 2:** Results of Fine–Gray model analysis for the cumulative incidence of relapse.

Factor	Hazard ratio (95% CI)	*P* value
Age ≥ 65 years	1.13 (0.68–1.87)	0.64
B symptoms	1.12 (0.68–1.85)	0.65
Bone marrow invasion	1.72 (1.03–2.88)	0.04
Extranodal lesions ≥ 2	1.07 (0.63–1.83)	0.80
Non GCB	2.21 (1.42–3.43)	< 0.001
PS ≥ 2	1.75 (0.78–3.92)	0.18
Post-treatment sIL-2R > UNL	2.30 (1.46–3.61)	< 0.001
Pre-treatment sIL2-R > UNL	1.05 (0.59–1.88)	0.86
Stage III or IV	2.82 (1.71–4.64)	< 0.001

*CI* confidence interval, *GCB* germinal centre B-cell-like type, *high-int* high-intermediate, *IPI* international prognostic index, *PS* performance status, *sIL-2R* soluble interleukin-2 receptor, *UNL* upper normal limit.

Furthermore, similar results were observed using the cut-off value for post-treatment sIL-2R level determined by ROC analysis, which was 504 U/mL (Supplementary Figure S3 online). Patients who had post-treatment sIL2R > 504 U/mL had significantly higher CIR compared with those who had post-treatment sIL2R ≤ 504 U/mL (Supplementary Figure S4a online) and the multivariable analysis showed the post-treatment sIL-2R level > 504 U/mL, non-GCB type, initial bone marrow invasion, and advanced stage at diagnosis to be independent prognostic factors for CIR (Supplementary Table S2 online).

### Subsequent survival

**Figure 2:**
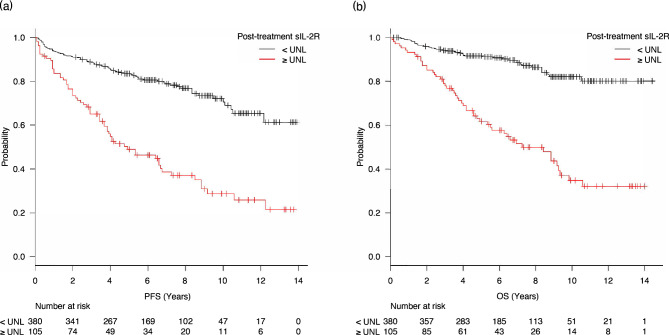
Progression-free survival and overall survival. Five-year PFS rate was significantly lower in the elevated sIL-2R group (49.0%; 95% CI, 38.4–58.6%) compared with the normal sIL-2R group (83.5%; 95% CI, 79.2–87.0%; *p* < 0.001). (**b**) Significantly inferior OS was observed in the elevated sIL-2R group with a five-year OS of 61.7% (95% CI, 50.9–70.8%) than in the normal sIL-2R group (five-year OS, 91.6%; 95% CI, 88.2–94.0%; *p* < 0.001). OS—overall survival; PFS—progression-free survival; sIL-2R—soluble interleukin-2 receptor; ULN—upper limit of normal.

Five-year PFS and OS in the overall cohort were 76.1% (95% CI, 71.9%–79.8%) and 85.1% (95% CI, 81.4%–88.1%), respectively. Five-year PFS was 49.0% (95% CI, 38.4%–58.6%) in the elevated sIL-2R group and 83.5% (95% CI, 79.2%–87.0%) in the normal sIL-2R group (HR, 3.47; 95% CI, 2.50–4.83; *p* < 0.001; Fig. 2a). In an analysis including only patients with elevated pre-treatment sIL-2R levels, PFS was shorter in patients with elevated post-treatment sIL-2R levels (five-year PFS 47.1%, 95% CI, 36.3%–57.2%) than those with normal post-treatment sIL-2R levels (five-year PFS 79.8%, 95% CI, 73.8%–84.7%, HR 2.87, 95% CI, 2.01–4.11, *p* < 0.001). Additionally, in a subgroup analysis limited to patients with limited-stage disease, five-year PFS was 66.2% (95% CI, 50.7% – 77.8%) for those with elevated post-treatment sIL-2R levels, and 89.0% (95% CI, 84.3% – 92.3%) for those with normal post-treatment sIL-2R levels (HR 3.84, 95% CI, 2.34–6.32, *p* < 0.001). Moreover, in patients with advanced-stage disease, the five-year PFS was 32.1% (95% CI, 19.2% – 45.8%) for those with elevated post-treatment sIL-2R levels, and 71.2% (95% CI, 61.7% – 78.8%) for those with normal post-treatment sIL-2R levels (HR 2.52, 95% CI, 1.62–3.93, *p* < 0.001).

Furthermore, the five-year OS rate was significantly lower in the elevated sIL-2R group (65.8%; 95% CI, 55.3%–74.4%) than in the normal sIL-2R group (91.5%; 95% CI, 88.1%–94.0%; HR, 4.85; 95% CI, 3.25–7.23; *p* < 0.001; Fig. 2b). This result remained consistent when an analysis was limited to patients with elevated pre-treatment sIL-2R levels. The five-year OS rate was 59.9% (95% CI, 48.6–69.4) in the elevated post-treatment sIL-2R group and 89.7% (95% CI, 84.7–93.1) in the normal post-treatment sIL-2R group (HR 4.14, 95% CI, 2.67–6.43, *p* < 0.001). The OS was associated with post-treatment sIL-2R levels in both limited and advanced stage patients: In patients with limited-stage disease, the five-year OS was 72.1% (95% CI, 56.8% – 82.8%) for those with elevated post-treatment sIL-2R levels, and 93.7% (95% CI, 89.7% – 96.1%) for those with normal post-treatment sIL-2R levels (HR 4.89 95% CI, 2.79–8.90, *p* < 0.001). In patients with advanced-stage disease, the five-year PFS was 32.1% (95% CI, 19.2% – 45.8%) for those with elevated post-treatment sIL-2R levels, and 71.2% (95% CI, 61.7% – 78.8%) for those with normal post-treatment sIL-2R levels (HR 3.79, 95% CI, 2.17–6.62, *p* < 0.001).

Notably, 35 PFS events occurred after five years, including 16 non-relapse deaths and 19 relapse events. The relapses were mostly systemic, including one patient each showing follicular lymphoma (FL), DLBCL with FL, and high-grade B-cell lymphoma with FL. Four patients experienced central nervous system relapses. The results of the univariable analyses of PFS and OS are shown in Supplementary Table S3 online. Multivariable analysis incorporating other established risk factors showed that age and post-treatment sIL-2R > ULN were prognostic factors for both PFS and OS. Additionally, non-GCB type and stage were prognostic of PFS, while B symptoms were prognostic of OS (Table 3, Supplementary Figure S5 and S6 online). The same factors remained prognostic of PFS and OS when the post-treatment sIL-2R cut-off value of 504 U/mL was applied (Supplementary Figure S4b,c, Supplementary Table S4 online).

**Table 3:** Results of Cox proportional hazards regression for PFS and OS.

Factor	PFS		OS	
	Hazard ratio	*P* value	Hazard ratio	*P* value
Age ≥ 65 years	1.68 (1.10–2.58)	0.02	6.65 (2.88–15.36)	< 0.001
B symptoms	1.26 (0.84–1.88)	0.26	1.67 (1.04–2.67)	0.03
Bone marrow invasion	1.31 (0.81–2.10)	0.27	1.14 (0.61–2.10)	0.68
Extranodal lesions ≥ 2	1.07 (0.66–1.74)	0.78	1.09 (0.59–2.03)	0.78
Non-GCB	1.54 (1.09–2.18)	0.01	1.14 (0.75–1.74)	0.53
PS ≥ 2	1.36 (0.69–2.68)	0.37	1.43 (0.67–3.04)	0.35
Post-treatment sIL-2R > UNL	2.57 (1.79–3.71)	< 0.001	3.15 (2.04–4.86)	< 0.001
Pre-treatment sIL-2R > UNL	1.25 (0.77–2.03)	0.37	1.36 (0.71–2.57)	0.35
Stage III or IV	2.02 (1.34–3.04)	0.01	1.49 (0.89–2.48)	0.13

*GCB* germinal centre B-cell-like type, *high-int* high-intermediate, *IPI* international prognostic index, *OS* overall survival, *PFS* progression-free survival, *PS* performance status, *sIL-2R* soluble interleukin-2 receptor, *UNL* upper normal limit.

## Discussion

In this study, we retrospectively analysed patients with DLBCL who achieved CMR after R-CHOP with or without RT as an initial treatment. To the best of our knowledge, this is the largest study with the longest follow-up period to date confirming that post-treatment sIL-2R levels are predictive of subsequent relapse, OS, and PFS in this patient cohort.

Among patients with DLBCL, the sIL-2R level at diagnosis has been previously reported to correlate with the IPI, metabolic tumour volume, and stage^26–28^. One group reported that serum sIL-2R levels decreased during complete remission and increased again during relapse or disease progression in non-Hodgkin lymphoma^27^. A retrospective study of 21 patients with newly diagnosed advanced-stage DLBCL reported that post-treatment sIL-2R levels were higher in patients who relapsed within 12 months than in those who did not^29^. Our analysis included patients with limited-stage and advanced-stage diseases who were shown to have achieved CMR using FDG-PET/CT after R-CHOP therapy. The CIR was higher in patients with elevated post-treatment serum sIL-2R levels than in those with normal sIL-2R levels. In addition, this group of patients exhibited lower PFS and OS rates. Our results suggest that patients with elevated post-treatment sIL-2R levels are at a high risk of relapse; therefore, we suggest more careful follow-up for this group of patients, including regular clinic visits in the first five years after treatment, in which the majority of relapse was observed, as well as patient education to detect any signs of relapse.

Furthermore, this group of patients may be possible candidates for future clinical trials evaluating consolidative or maintenance therapies to reduce the risk of relapse. Notably, although more patients in the elevated sIL-2R group than in the normal sIL-2R group had elevated pre-treatment sIL-2R levels, post-treatment sIL-2R, but not pre-treatment sIL-2R, was independently prognostic of subsequent relapse, PFS, and OS in multivariable analysis. These results suggest that post-treatment sIL-2R levels predict subsequent relapse and survival more accurately than pre-treatment sIL-2R levels.

FDG-PET/CT is currently the gold standard for evaluating treatment responses in FDG-avid lymphomas, including DLBCL. However, 7%–20% of patients with CMR experience relapse^30^. In our study, 17.5% of patients experienced relapse within 5 years, which is comparable to the findings of previous reports. One possible explanation for the correlation between post-treatment sIL-2R levels and subsequent relapse in patients with negative end-of-treatment FDG-PET/CT findings is that sIL-2R may reflect an infinitesimal amount of residual disease that could not be detected by FDG-PET/CT. Thus, the incorporation of post-treatment sIL-2R along with FDG-PET/CT assessment may improve the prognostic accuracy in newly diagnosed DLBCL patients.

A subset of large B-cell lymphomas containing the translocation of *MYC* genes with *BCL-2* and/or *BCL-6* genes is now recognised as a separate entity, high-grade B-cell lymphoma^2^. This group of lymphomas has inferior survival^31–33^. Furthermore, several studies have reported that concurrent MYC and BCL-2 expression in DLBCL is associated with a lower complete response rate as well as shorter PFS and OS^34–36^. We were unable to obtain sufficient data on MYC expression and IgH-*MYC* and IgH-*BCL2* translocations. Further investigation is warranted regarding the influence of double-hit status or double-expressor status on post-treatment sIL-2R levels and subsequent relapse.

There are no internationally established uniform methods for analyzing serum sIL-2R, and the cut-off value differs in each method used. Therefore, we evaluated both the “upper limit of normal”, which is 519 U/mL for the method we used, and the value determined by ROC analysis of our cohort, which was 504 U/mL, as the cut-off for post-treatment serum sIL-2R. Although the exact cut-off value is yet to be validated, similar results were obtained using the above cut-off values, suggesting these values can be used to identify DLBCL patients treated with R-CHOP and who achieved CMR, that are at higher risk of relapse.

The limitations of this study are as follows. First, it was conducted at a single institution, which potentially limited the generalisability of the results. Second, owing to the retrospective nature of this study, we were unable to eliminate possible bias; thus, the results should be interpreted with caution. Third, the treatment evaluation did not follow a uniform time point as it was determined by each treating physician. Although it was generally conducted within the recommended time frame of 6–8 weeks after completion of chemotherapy and 8–12 weeks after completion of radiotherapy, the timing of FDG-PET/CT may have affected the response evaluation. Lastly, patients without end-of-treatment FDG-PET/CT data and those with concomitant low-grade lymphoma were excluded from the analysis; therefore, the significance of post-treatment serum sIL-2R levels in predicting subsequent relapse in these groups of patients remains to be investigated. Nevertheless, we demonstrated novel findings suggesting that post-treatment sIL-2R is a sensitive prognostic marker in patients with uniformly treated newly diagnosed DLBCL who achieved CMR.

In conclusion, in patients with newly diagnosed DLBCL who achieved CMR after treatment with R-CHOP, the post-treatment serum sIL-2R level was an independent prognostic marker of subsequent relapse as well as PFS and OS, along with other factors previously known to predict survival in lymphoma patients.

### Supplementary Information

Supplementary Information.

## Data Availability

The data that support the findings of this study are available from the corresponding author upon request.
