# Supplementary material for: Prognostic value of post-treatment serum soluble interleukin-2 receptor in newly diagnosed diffuse large B-cell lymphoma patients who achieved complete metabolic response following R-CHOP therapy
Source: Sci Rep. 2023 Aug 22;13:13713. doi: 10.1038/s41598-023-40026-7 (PMC10444791; doi:10.1038/s41598-023-40026-7)
Supplement: Supplementary file 1 — Supplementary Information. [file 41598_2023_40026_MOESM1_ESM.pdf]

Supplementary Information

Prognostic value of post-treatment serum soluble interleukin-2 receptor in newly diagnosed diffuse large B-cell lymphoma patients who achieved complete metabolic response following R-CHOP therapy

Yuko Shirouchi[1], Noriko Nishimura[1], Yuko Mishima[1], Yuko Ishihara[1], Hiroaki Asai[1], Mikako Tamba[1], Mitsuhito Hirano[1], Kei Hirano[1], Yukako Teramoto[1], Kikuaki Yoshida[1], Kengo Takeuchi[2][3][4], Takashi Terauchi[5], and Dai Maruyama[1]

[1] *Department of Hematology Oncology, Cancer Institute Hospital, Japanese Foundation for Cancer Research, Tokyo, Japan*

[2] *Division of Pathology, The Cancer Institute, Japanese Foundation for Cancer Research, Tokyo, Japan*

[3] *Pathology Project for Molecular Targets, The Cancer Institute, Japanese Foundation for Cancer Research, Tokyo, Japan*

[4] *Department of Pathology, Cancer Institute Hospital, Japanese Foundation for Cancer Research, Tokyo, Japan*

[5] *Department of Nuclear Medicine, Cancer Institute Hospital, Japanese Foundation for Cancer Research, Tokyo, Japan*

Corresponding Author; Dai Maruyama (Email: dai.maruyama@jfcr.or.jp)

Supplementary Table S1. Results of Gray's test for the cumulative incidence of relapse

Factor	Group	n	5-year PFS	*p*-value
Age (years)	<65	169	14.8% (9.8–20.8%)	
	≥65	316	20.3% (15.9–25.1%)	0.091
B symptoms	yes	81	34.2% (23.9–44.8%)	
	no	404	15.2% (11.8–19.1%)	< 0.001
Bone marrow invasion	yes	73	36.7% (25.5–48.0%)	
	no	412	15.1% (11.7–18.9%)	< 0.001
Extranodal lesions	0 or 1	396	14.3% (10.9–18.1%)	
	>1	89	37.0% (26.6–47.5%)	< 0.001
IPI	low/low-int	358	11.8% (8.6–15.6%)	
	high/high-int	127	37.4% (28.5–46.3%)	< 0.001
COO	GCB	251	10.2% (6.8–14.6%)	
	non-GCB	220	28.3% (22.3–34.6%)	< 0.001
PS	0 or 1	464	16.9% (13.6–20.6%)	
	>1	21	51.4% (25.8–72.1%)	< 0.001
Post-treatment sIL-2R	≤UNL	380	12.8% (9.6–16.5%)	
	>UNL	105	38.8% (29.0–48.5%)	< 0.001
Pre-treatment sIL-2R	≤UNL	163	9.2% (5.3–14.5%)	
	>UNL	322	23.0% (18.4–28.0%)	< 0.001
Stage	I or II	311	9.2% (6.2–12.9%)	
	III or IV	174	35.1% (27.65–42.5%)	< 0.001

COO, cell of origin; high-int, high-intermediate; IPI, international prognostic index; low-int, low-intermediate; PFS, progression-free survival; PS, performance status; sIL-2R, soluble interleukin-2 receptor; UNL, upper normal limit.

Supplementary Table S2. Results of Fine–Gray model analysis for the cumulative incidence of relapse using the cut-off post-treatment sIL-2R value of 504 U/mL

Factor	Hazard ratio (95% CI)	P value
Age ≥ 65 years	1.10 (0.66–1.84)	0.70
B symptoms	1.14 (0.70–1.87)	0.59
Bone marrow invasion	1.66 (0.99–2.78)	0.05
Extranodal lesions ≥ 2	1.06 (0.62–1.81)	0.83
Non GCB	2.17 (1.39–3.38)	< 0.001
PS ≥ 2	1.81 (0.80–4.08)	0.15
Post-treatment sIL-2R > 504 U/mL	2.13 (1.36–3.34)	< 0.001
Pre-treatment sIL2-R > UNL	1.06 (0.59–1.89)	0.86
Stage III or IV	2.90 (1.77–4.75)	< 0.001

CI, confidence interval; GCB, germinal centre B-cell-like type; high-int, high-intermediate; IPI, international prognostic index; PS, performance status; sIL-2R, soluble interleukin-2 receptor; UNL, upper normal limit

Supplementary Table S2. Univariable analysis for PFS and OS

Factor	Group	n	5-year PFS	*p*-value	5-year OS	*p*-value
Age (years)	<65	169	83.8% (77.1–88.7%)		96.1% (91.4–98.2%)	
	≥65	316	73.7% (68.2-78.4%)	<0.001	79.3% (74.1–83.6%)	<0.001
B symptoms	yes	81	55.9% (44.0–66.3%)		65.9% (54.0–75.4%)	
	no	404	80.2% (75.7–83.9%)	<0.001	89.0% (85.3–91.8%)	<0.001
Bone marrow invasion	yes	73	61.1% (48.4–71.6%)		79.2% (67.3–87.2%)	
	no	412	88.8% (74.3–82.6%)	0.002	86.2% (82.2–89.3%)	0.11
Extranodal lesions	0 or 1	396	80.3% (75.8–84.0%)		87.6% (83.7–90.6%)	
	>1	89	56.8% (45.1–67.0%)	<0.001	74.0% (62.8–82.3%)	<0.001
IPI	low/low-int	358	83.1% (78.6–86.7%)		89.9% (86.1–92.8%)	
	high/high-int	127	65.6% (45.7–64.3%)	<0.001	71.1% (61.6–78.6%)	<0.001
COO	GCB	251	84.1% (78.7–88.3%)		89.6% (84.8–92.9%)	
	non-GCB	220	66.0% (59.0–72.1%)	<0.001	79.7% (73.3–84.7%)	0.11
PS	0 or 1	464	77.6% (73.3–81.3%)		86.0% (82.3–89.0%)	
	>1	21	42.4% (19.7–63.6%)	<0.001	65.5% (40.6–82.0%)	<0.001
Post-treatment sIL-2R	≤UNL	380	83.5% (79.2–87.0%)		91.6% (88.2%–94.0%)	
	>UNL	105	49.0% (38.4–58.6%)	<0.001	61.7% (50.9–70.8%)	<0.001
Pre-treatment sIL-2R	≤UNL	163	87.9% (81.5–92.2%)		93.9% (88.6–96.8%)	
	>UNL	322	70.1% (64.5–75.0%)	<0.001	80.6% (75.5–84.7%)	<0.001
Stage	I or II	311	85.4% (80.7–88.9%)		90.2% (86.1–93.1%)	
	III or IV	174	59.1% (50.9–66.5%)	<0.001	76.0% (68.4–82.0%)	<0.001

COO, cell of origin; high-int, high-intermediate; IPI, international prognostic index; low-int, low-intermediate; OS, overall survival; PFS, progression-free survival; PS, performance status; sIL-2R, soluble interleukin-2 receptor; UNL, upper normal limit

Supplementary Table S4. Results of Cox proportional hazards regression for PFS and OS using the cut-off post-treatment sIL-2R value of 504 U/mL

	PFS		OS	
Factor	Hazard ratio	P value	Hazard ratio	P value
Age ≥ 65 years	1.64 (1.07–2.53)	0.02	6.50 (2.81–15.01)	< 0.001
B symptoms	1.26 (0.84–1.89)	0.26	1.65 (1.03–2.64)	0.04
Bone marrow invasion	1.25 (0.77–2.02)	0.36	1.07 (0.58–2.00)	0.82
Extranodal lesions ≥ 2	1.08 (0.66–1.76)	0.77	1.15 (0.62–2.15)	0.65
Non-GCB	1.52 (1.08–2.15)	0.02	1.14 (0.75–1.74)	0.54
PS ≥ 2	1.43 (0.73–2.82)	0.30	1.50 (0.70–3.19)	0.29
Post-treatment sIL-2R > 504 U/mL	2.36 (1.64–3.40)	< 0.001	2.65 (1.71–4.10)	< 0.001
Pre-treatment sIL-2R > UNL	1.25 (0.77–2.04)	0.37	1.43 (0.75–2.70)	0.28
Stage III or IV	2.07 (1.373.13)	< 0.001	1.50 (0.90–2.51)	0.12

GCB, germinal centre B-cell-like type; high-int, high-intermediate; IPI, international prognostic index; OS, overall survival; PFS, progression-free survival; PS, performance status; sIL-2R, soluble interleukin-2 receptor; UNL, upper normal limit

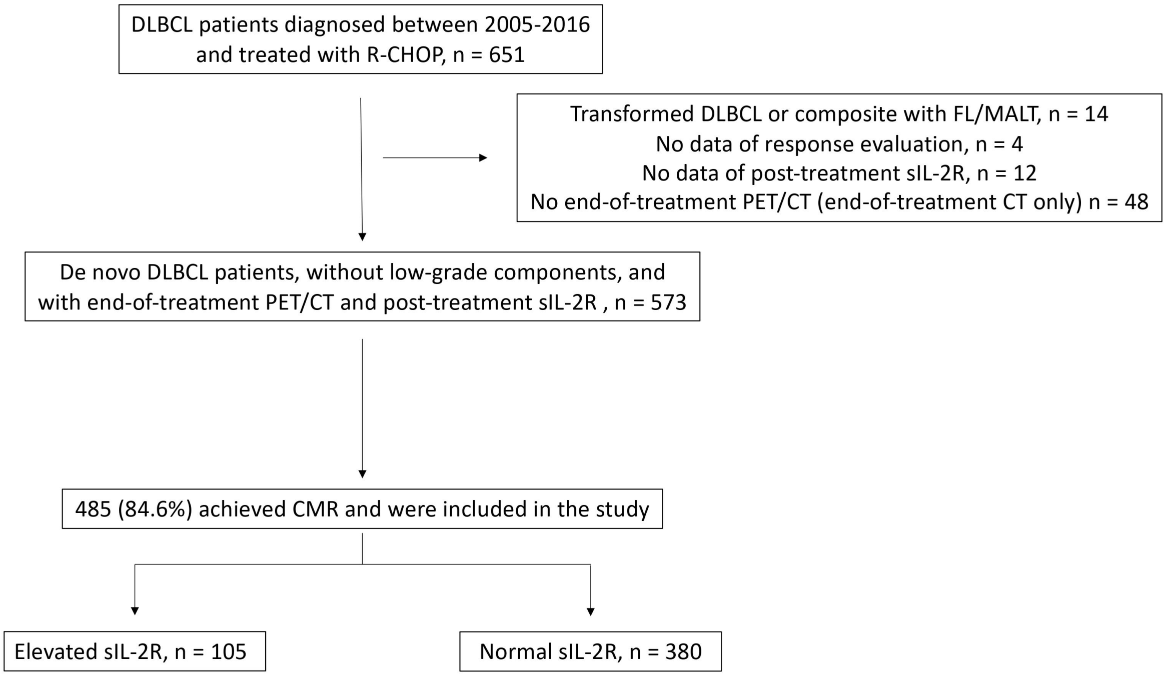

Supplementary Fig. S1 - CONSORT diagram of the study

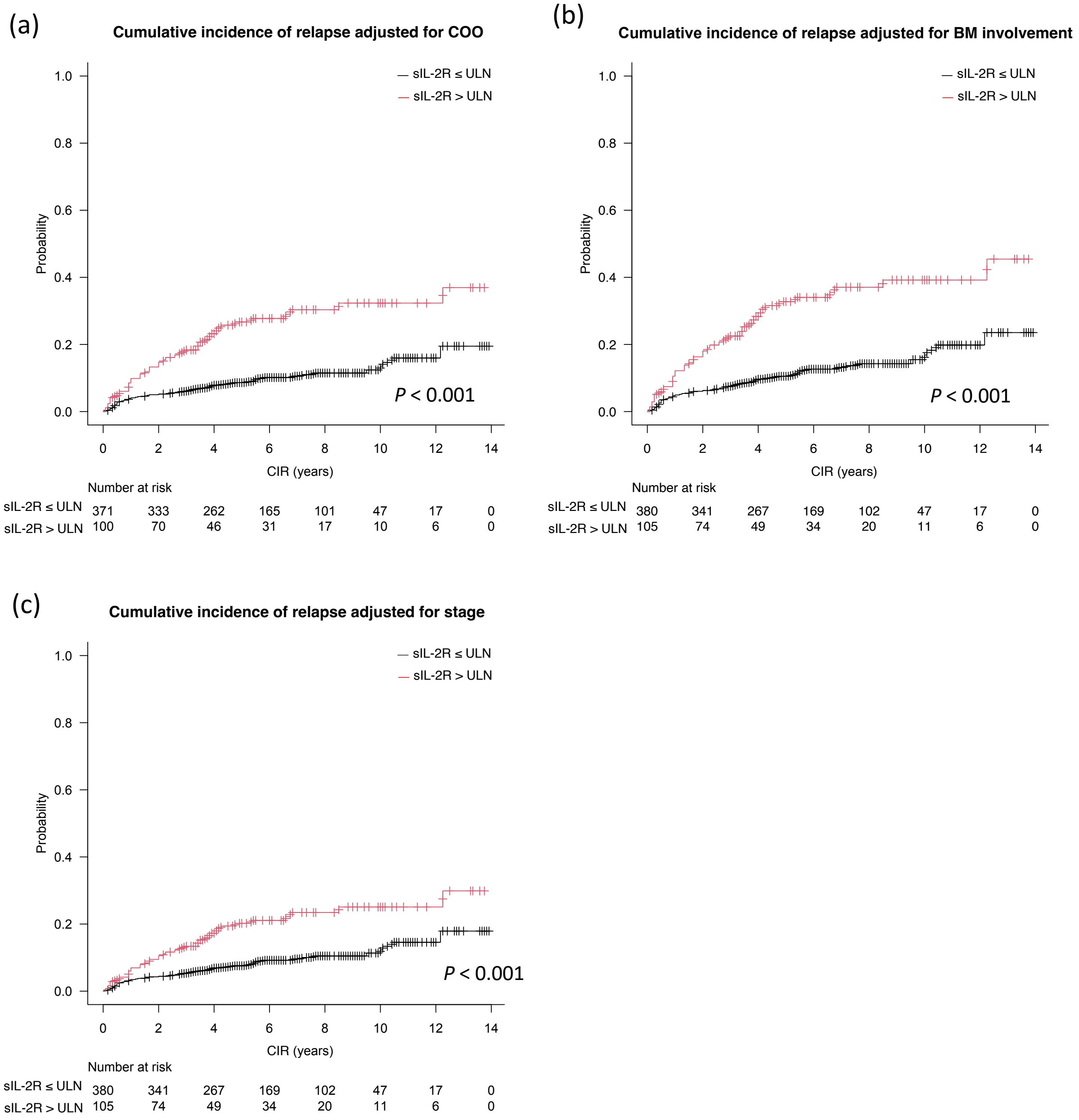

Supplementary Fig. S2. Cumulative incidence of relapse adjusted for (a) COO, (b) initial bone marrow involvement, and (c) stage at diagnosis.

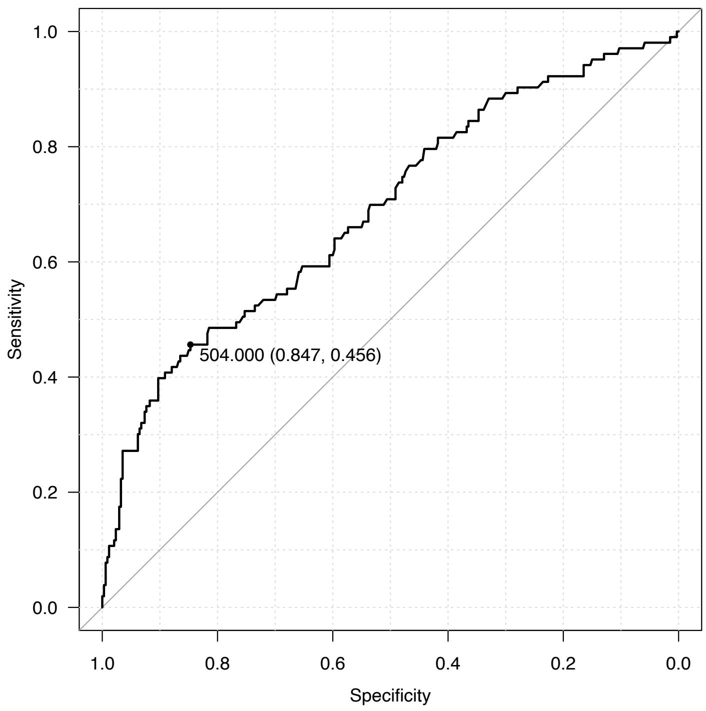

Supplementary Fig. S3. ROC curve analysis for post-treatment sIL-2R level. Area under the curve 0.69 (95% CI 0.629 – 0.751).

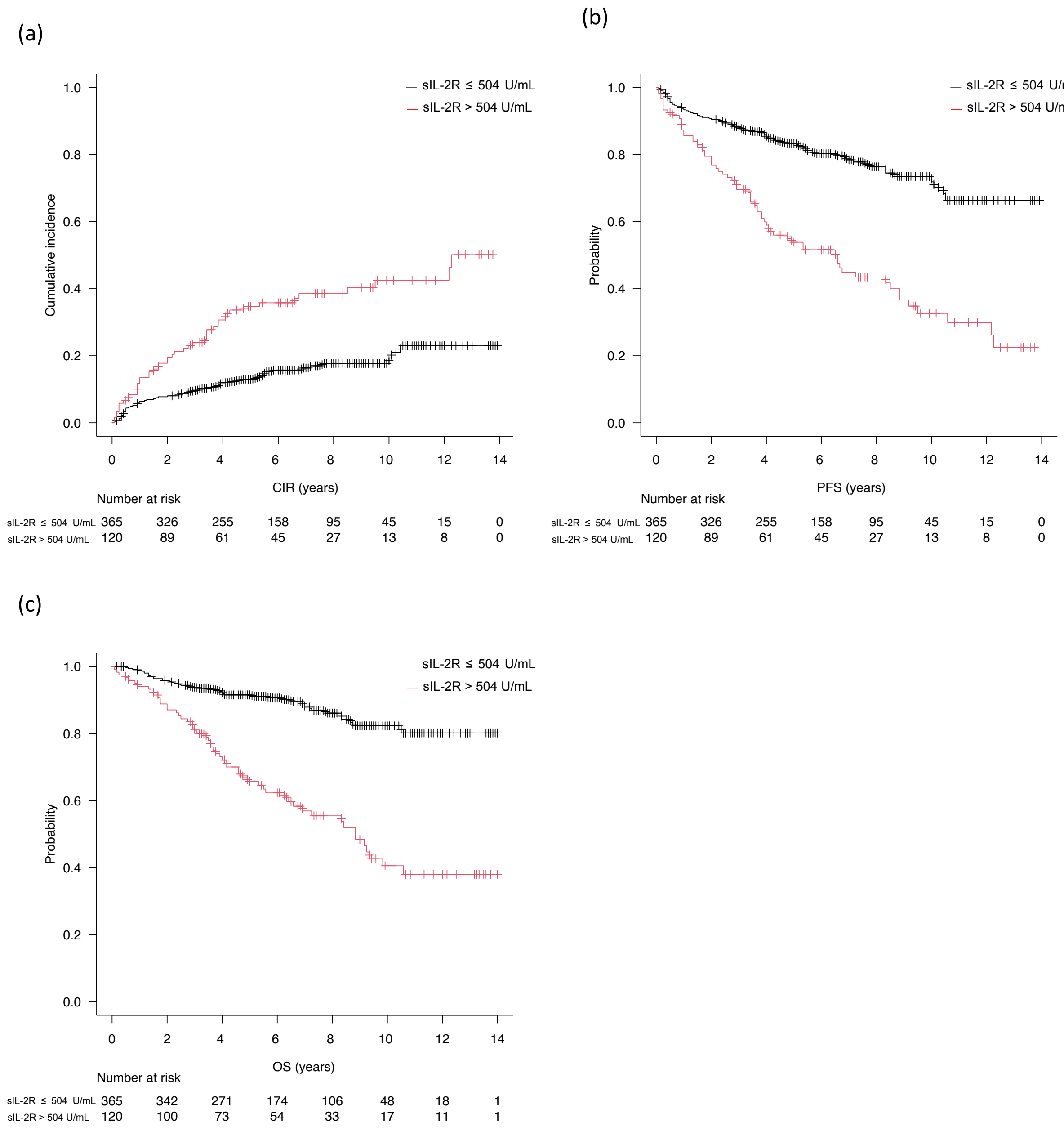

Supplementary Fig. S4. (a) Cumulative incidence of relapse (b) progression-free survival, and (c) overall survival for patients with post-treatment sIL-2R ≤ 504 U/mL and those with post-treatment sIL-2R > 504 U/mL.

(a) Significantly higher CIR was observed in patients with post-treatment sIL2R > 504 U/mL (five-year CIR, 34.7%; 95% CI, 25.9–43.6%) compared with patients with post-treatment sIL2R ≤ 504 U/mL (five-year CIR, 13.0%; 95% CI, 9.7–16.8; p < 0.001).
(b) Five-year PFS rate was significantly lower in patients with post-treatment sIL2R > 504 U/mL (53.8%; 95% CI, 44.4–62.7%) compared with those with post-treatment sIL2R ≤ 504 U/mL (83.4%; 95% CI, 79.0–87.0%; p < 0.001).
(c) Five-year OS rate was significantly lower in patients with post-treatment sIL2R > 504 U/mL (65.7%; 95% CI, 55.8–73.9%) compared with those with post-treatment sIL2R ≤ 504 U/mL (91.5%; 95% CI, 88.0–94.1%; p < 0.001).

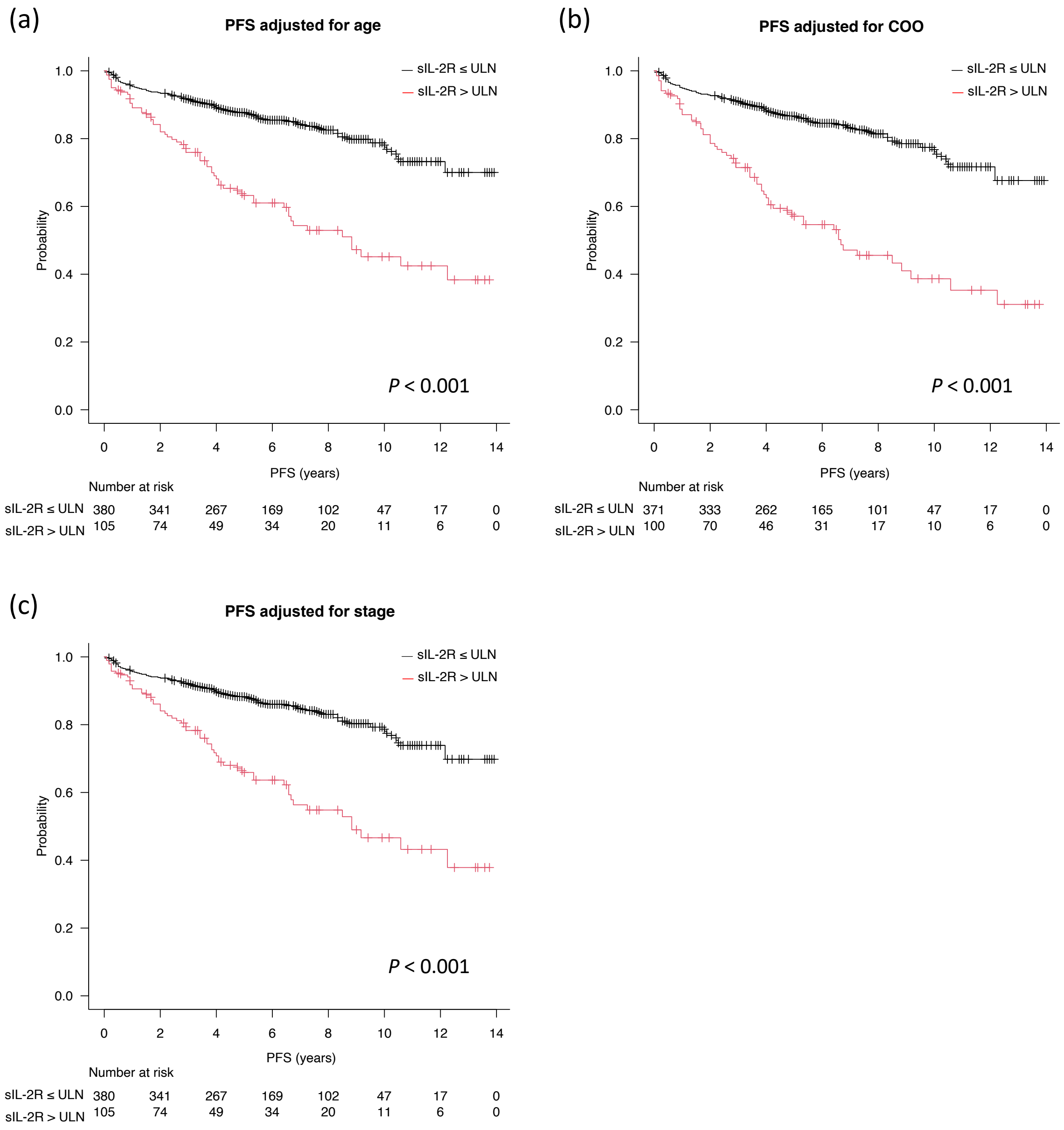

Supplementary Fig. S5. Progression-free survival adjusted for (a) age, (b) cell of origin, and (c) stage at diagnosis.

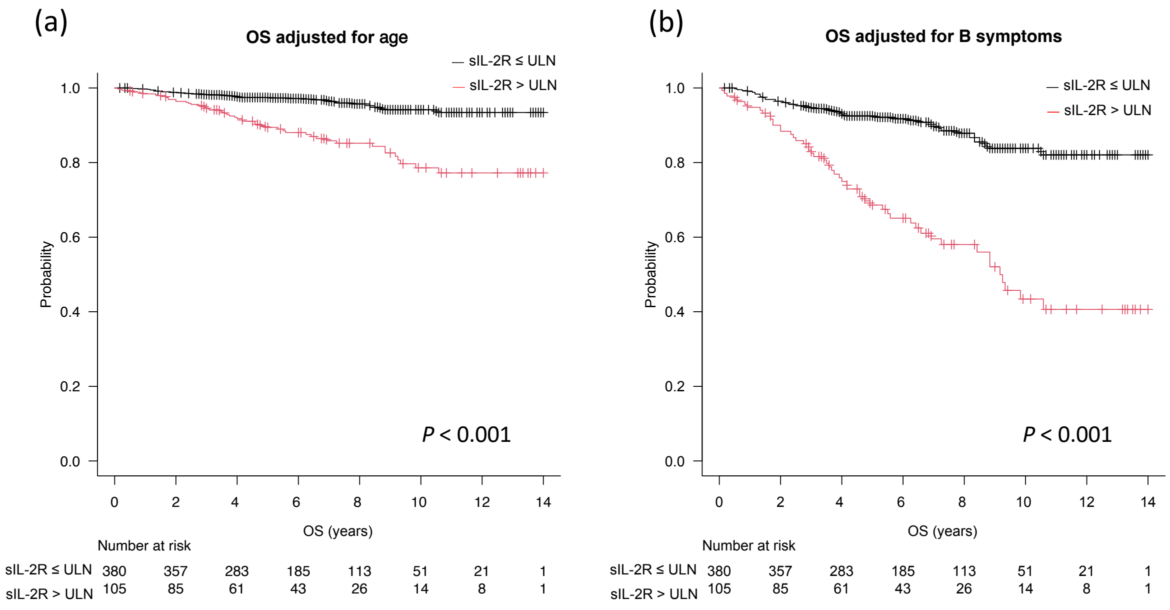

Supplementary Fig. S6. Overall survival adjusted for (a) age and (b) the presence or absence of B symptoms.
